# Overexpression of long noncoding RNA ANRIL inhibits phenotypic switching of vascular smooth muscle cells to prevent atherosclerotic plaque development *in vivo*

**DOI:** 10.18632/aging.202392

**Published:** 2020-12-19

**Authors:** Da-Jun Hu, Zhen-Yu Li, Yuan-Ting Zhu, Chuan-Chang Li

**Affiliations:** 1 Department of Geriatric Medicine, Xiangya Hospital, Central South University, Changsha 410008, China; 2 Department of Cardiology, The First People's Hospital of Chenzhou, Chenzhou 423000, China; 3 National Clinical Research Center for Geriatric Disorders, Xiangya Hospital, Central South University, Changsha 410008, China

**Keywords:** long non-coding RNA (lncRNA), ANRIL, AMP-activated protein kinase, metformin, atherosclerosis

## Abstract

Background: Phenotypic switching of vascular smooth muscle cells (VSMCs) plays a key role in atherosclerosis. Long noncoding RNA ANRIL (lncRNA-ANRIL) is critical in vascular homeostasis. Metformin produces multiple beneficial effects in atherosclerosis. However, the underlying mechanisms need to be elucidated.

Methods and Results: Metformin increased lncRNA-ANRIL expression and AMPK activity in cultured VSMCs, and inhibited the phenotypic switching of VSMCs to the synthetic phenotype induced by platelet-derived growth factor (PDGF). Overexpression of lncRNA-ANRIL inhibited phenotypic switching and reversed the reduction of AMPK activity in PDGF-treated VSMCs. While, gene knockdown of lncRNA-ANRIL by adenovirus or silence of AMPKγ through siRNA abolished AMPK activation induced by metformin in VSMCs. RNA-immunoprecipitation analysis indicated that the affinity of lncRNA-ANRIL to AMPKγ subunit was increased by metformin. *In vivo*, administration of metformin increased the levels of lncRNA-ANRIL, suppressed VSMC phenotypic switching, and prevented the development of atherosclerotic plaque in *Apoe^-/-^* mice fed with western diet. These protective effects of metformin were abolished by infecting *Apoe^-/-^* mice with adenovirus expressing lncRNA-ANRIL shRNA. The levels of AMPK phosphorylation, AMPK activity, and lncRNA-ANRIL expression were decreased in human atherosclerotic lesions. Conclusion: Metformin activates AMPK to suppress the formation of atherosclerotic plaque through upregulation of lncRNA-ANRIL.

## INTRODUCTION

It is known that atherosclerosis is a chronic inflammation in arterial wall [1, 2]. Vascular smooth muscle cells (VSMCs) can change the phenotype from contractile to synthetic as a response to multiple stimuli in atherosclerosis [3, 4]. Further characterization and better understanding of the underlying signaling could provide a therapeutic target in the prevention of atherosclerosis.

Protein function can be regulated by RNA-protein interaction [5, 6]. Long noncoding RNA (LncRNA) is important to regulate cellular functions including translation, transcription, and cell differentiation [7, 8]. The ANRIL gene encoding a 3.8 kb lncRNA, consisting of 19 exons, is robustly expressed in vascular cells [9]. Human studies showed that lncRNA-ANRIL expression is reduced in coronary artery samples [10–12]. It is also reported that lncRNA-ANRIL regulates endothelial cell and VSMCs functions by transcriptionally upregulating several genes expressions [13, 14], revealing the critical roles of lncRNA-ANRIL in controlling vascular functions.

AMP-activated protein kinase (AMPK) is composed of α, β, and γ subunits [15]. The α subunit is a catalytic subunit, while β and γ subunits are regulatory subunits [16, 17]. An increased AMP level is able to activate AMPK through allosteric effect to maintain AMPKα phosphorylation within eukaryotic cells [18]. AMPK is also activated by serval drugs such as metformin, which exerts protective effects and affects VSMC phenotypic switching in vascular cells [19–21].

Based on the aforementioned studies, we speculated that, in VSMCs, lncRNA-ANRIL is a regulator of AMPK to inhibit phenotypic switching. The present study was aimed to establish the link between lncRNA-ANRIL and AMPK, and to test whether metformin prevents atherosclerosis through lncRNA-ANRIL/AMPK signaling.

## RESULTS

### PDGF reduces lncRNA-ANRIL level and increases phenotypic switching in VSMCs

**Figure 1:**
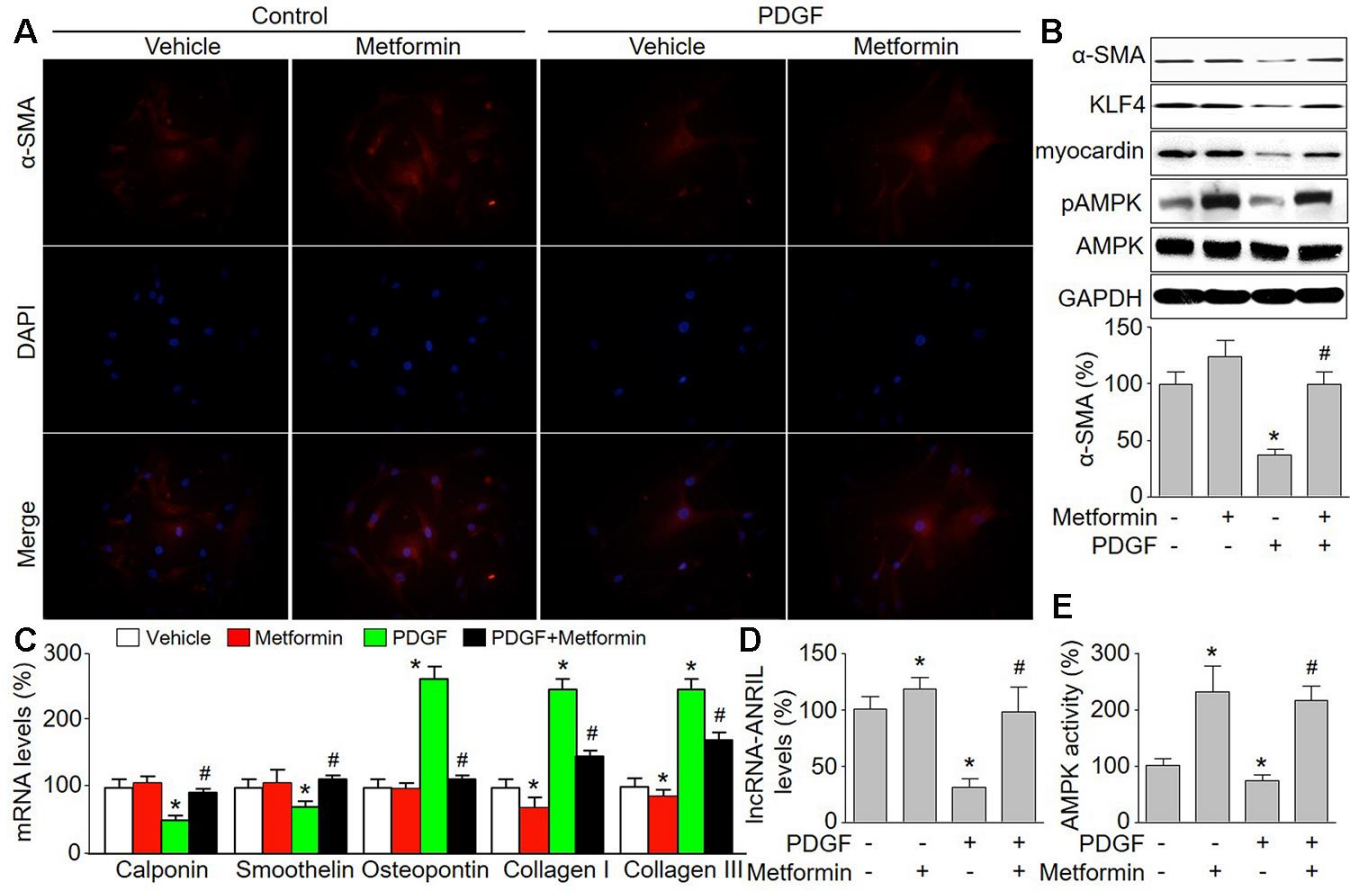
**Metformin inhibits PDGF-induced phenotypic switching and increases the expressions of lncRNA-ANRIL in cultured VSMCs.** Cultured VSMCs were treated with PDGF (5 ng/ml) for 48 hours in presence or absence of metformin (1 mM). (**A**) The morphology of contractile phenotype in cells was determined by immunofluorescence analysis of alpha SMA (α-SMA). (**B**) Total cell lysates were subjected to perform western blot analysis of phosphorylated AMPK (pAMPK), total AMPK protein levels, α-SMA, KLF4, and myocardin. (**C**) The mRNA levels of the phenotypic switching markers, including calponin, smoothelin, osteopontin, collagen I, and collagen III were measured by real-time PCR. (**D**) The lncRNA-ANRIL level was assessed by real-time PCR. (**E**) AMPK activity was assayed by P^32^-ATP method. N is 5 in each group. ^*^P<0.05 vs. Vehicle. ^#^*P*<0.05 vs. PDGF.

VSMC phenotypic switching is vital in the formation of atherosclerotic plaque [22]. Platelet-derived growth factor (PDGF) can promote VSMCs switching from the contractile phenotype to synthetic phenotype, which contributes to atherosclerosis [23]. We determined the effects of PDGF on lncRNA-ANRIL *DQ485454* transcript expression, which the expression level of the *DQ485454* transcript was >6-fold higher in atherosclerosis plaque than the levels of full-length *NR*_*003529* transcript or *EU741058* transcript as reported by Hyosuk Cho et al [13]. Phenotypic switching was detected by using IFC staining of α-SMA. LncRNA-ANRIL expression was assayed by real-time PCR. As indicated in Figure 1A, 1B, PDGF decreased α-SMA, KLF4, and myocardin protein levels, compared to vehicle-treated cells. The mRNA levels of contractile phenotypic markers, such as calponin and smoothelin, were also reduced in PDGF-treated cells (Figure 1C). Gene expressions of synthetic phenotypic markers, including osteopontin, collagen I, and collagen III, were upregulated by PDGF. Besides, the mRNA levels of lncRNA-ANRIL were reduced by PDGF in VSMCs (Figure 1D). These data indicate that PDGF induced phenotypic switching of VSMCs, consistent with other reports [24, 25].

### Metformin upregulates lncRNA-ANRIL expression and inhibits PDGF-induced phenotypic switching in VSMCs

Metformin has been reported to activate AMPK and is prescribed worldwide as anti-diabetic drug to manage type 2 diabetes [26]. Epidemiological evidence has demonstrated metformin is more likely to reduce cardiovascular event risk [27]. Thus, we determined the effects of metformin on PDGF-induced phenotypic switching in VSMCs. As shown in Figure 1B, 1E, metformin at 1 mM activated AMPK by increasing AMPK phosphorylation at Thr172, which is essential for AMPK activity [28]. As expected, metformin treatment abolished PDGF-induced the reductions of α-SMA, KLF4, and myocardin (Figure 1B). Metformin increased other contractile phenotypic markers (calponin and smoothelin) but decreased synthetic phenotypic markers (osteopontin, collagen I, and collagen III) in PDGF-treated VSMCs (Figure 1C). These data demonstrate that metformin is able to inhibit phenotypic switching of VSMCs. Further, metformin abrogated PDGF-induced reduction of lncRNA-ANRIL expression in VSMCs (Figure 1D).

### Overexpression of lncRNA-ANRIL abolishes PDGF-induced phenotypic switching

**Figure 2:**
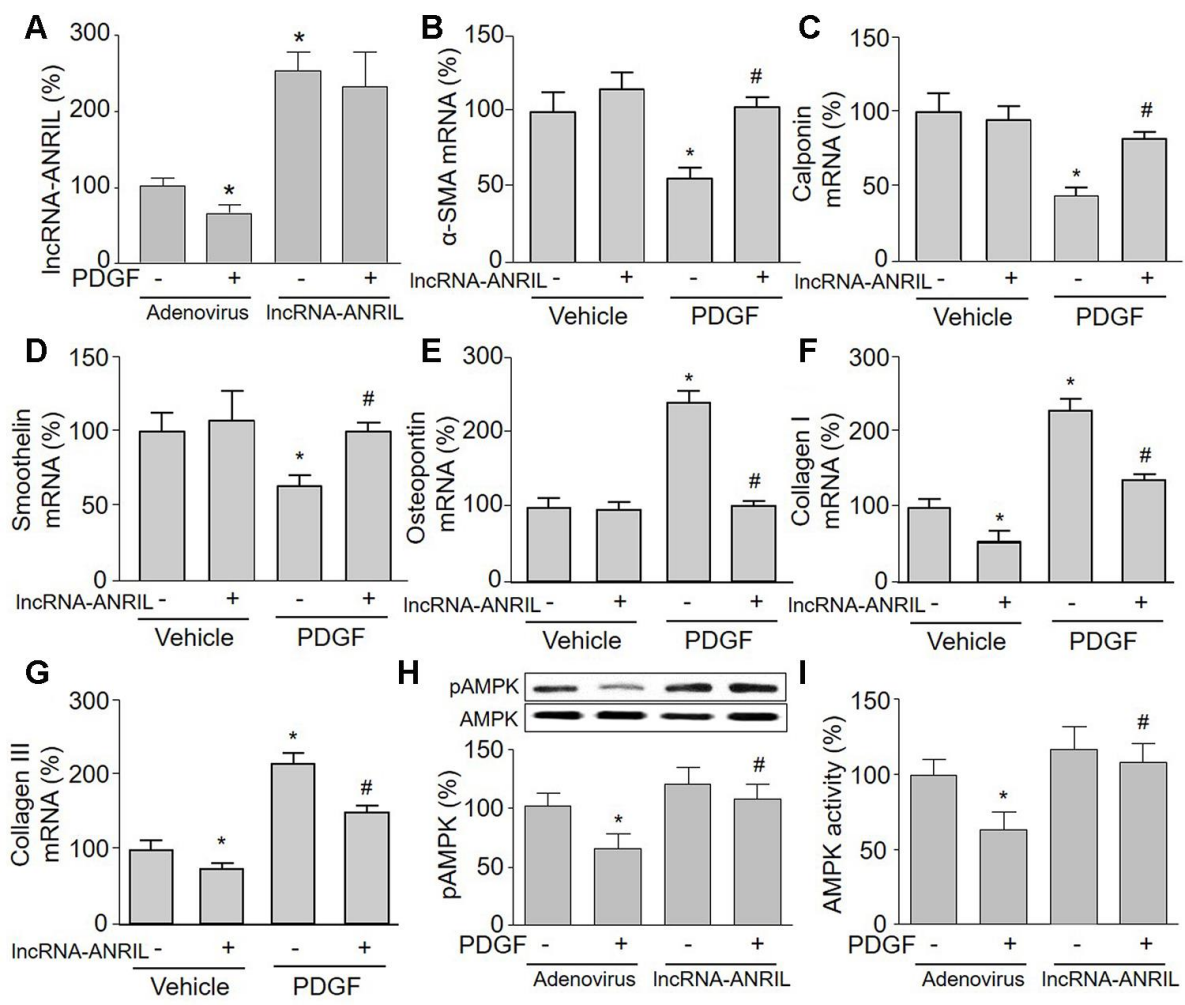
**Overexpression of lncRNA-ANRIL abolishes PDGF-induced phenotypic switching and reverses AMPK activity in cultured VSMCs.** Cultured VSMCs were infected with adenovirus expressing lncRNA-ANRIL for 24 hours and then treated with PDGF (5 ng/ml) for 48 hours. (**A**) The expression of lncRNA-ANRIL was determined by real-time PCR. N is 5 in each group. ^*^P<0.05 vs. adenovirus vector. (**B**–**G**) The mRNA levels of the markers of VSMC phenotypic switching, including α-SMA in B, calponin in C, smoothelin in D, osteopontin in E, collagen I in F, and collagen III in G were measured by real-time PCR. (**H**) Total cell lysates were subjected to perform western blot analysis of phosphorylated AMPK (pAMPK) and total AMPK protein levels. (**I**) AMPK activity was assayed by P^32^-ATP method. N is 5 in each group. ^*^P<0.05 vs. adenovirus alone. ^#^*P*<0.05 vs. adenovirus + PDGF.

To investigate the role of lncRNA-ANRIL in PDGF-induced phenotypic switching, we upregulated lncRNA-ANRIL in VSMCs via adenovirus, which PDGF had no effects on exogenous expression of lncRNA-ANRIL (Figure 2A). As depicted in Figure 2B–2G, in PDGF-treated cells, lncRNA-ANRIL overexpression reversed the expressions of contractile phenotypic markers (α-SMA, calponin, and smoothelin), compared with cells infected with adenovirus alone. However, lncRNA-ANRIL gain-function decreased mRNA levels of synthetic phenotypic markers (osteopontin, collagen I, and collagen III) in PDGF-treated VSMCs. These data demonstrate that PDGF via downregulation of lncRNA-ANRIL induces VSMC phenotypic switching.

### Gain-function of lncRNA-ANRIL increases AMPK activity in PDGF-treated VSMCs

To test whether AMPK is involved in PDGF-induced VSMC phenotypic switching via lncRNA-ANRIL dysfunction, we next examined AMPK phosphorylation and activity in lncRNA-ANRIL-overexpressed cells. As presented in Figure 2H, 2I, PDGF significantly inactivated AMPK by decreasing AMPK phosphorylation and its activity in cells infected with adenovirus vector alone, but had no effects on AMPK activity and phosphorylation if cells were overexpressed with lncRNA-ANRIL.

### Knockdown of lncRNA-ANRIL ablates pharmacological activations of AMPK in cultured VSMCs

**Figure 3:**
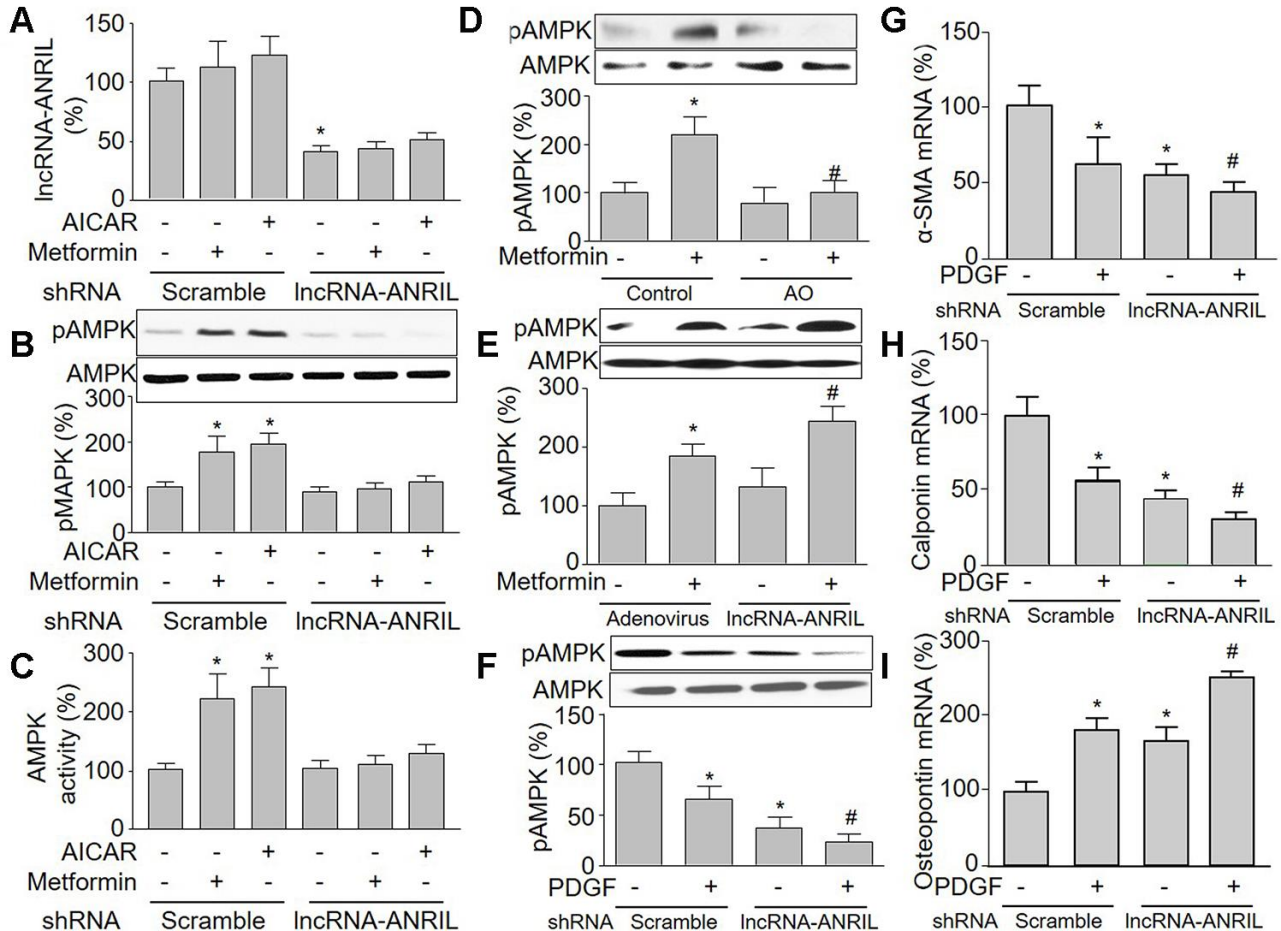
**Knockdown of lncRNA-ANRIL ablates pharmacological activations of AMPK in cultured VSMCs.** (**A**–**C**) Cultured VSMCs were infected with adenovirus expressing scramble or lncRNA-ANRIL shRNA for 48 hours and then treated with metformin (1 mM) or AICAR (0.5 mM) for 6 hours. (**A**) The expression of lncRNA-ANRIL was determined by real-time PCR. N is 5 in each group. ^*^P<0.05 vs. adenovirus. (**B**) Total cell lysates were subjected to perform western blot analysis of phosphorylated AMPK (pAMPK) and total AMPK protein levels. (**C**) AMPK activity was assayed by P^32^-ATP method. N is 5 in each group. ^*^P<0.05 vs. scramble shRNA alone. ^#^*P*<0.05 vs. scramble shRNA plus metformin or AICAR. (**D**) Cultured VSMCs were transfected antisense oligonucleotide of lncRNA-ANRIL (AO) for 48 hours and then treated with metformin (1 mM) for 6 hours. The pAMPK level was assayed by western blot in total cell lysates. N is 5 in each group. ^*^P<0.05 vs. control. ^#^*P*<0.05 vs. metformin alone. (**E**) Cultured VSMCs were infected with adenovirus expressing lncRNA-ANRIL for 24 hours and then treated with metformin (1 mM) for 6 hours. The pAMPK level was assayed by western blot in total cell lysates. N is 5 in each group. ^*^P<0.05 vs. adenovirus alone. ^#^*P*<0.05 vs. adenovirus plus metformin. (**F**–**I**) Cultured VSMCs were infected with adenovirus expressing lncRNA-ANRIL shRNA for 24 hours and then treated with PDGF (5 ng/ml) for 48 hours. Total cell lysates were subjected to perform western blot analysis of pAMPK in F. The mRNA levels of the phenotypic switching markers, including α-SMA in (**G**), calponin in (**H**), and osteopontin in I were assessed by real-time PCR. N is 5 in each group. ^*^P<0.05 vs. scramble shRNA alone. ^#^*P*<0.05 vs. lncRNA-ANRIL shRNA alone.

To further confirm whether lncRNA-ANRIL is an upstream modulator of AMPK in VSMCs, cells were infected with shRNA to downregulate lncRNA-ANRIL expression and then treated with metformin. Adenovirus-mediated shRNA expression inhibited lncRNA-ANRIL expression (Figure 3A). As indicated in Figure 3B, 3C, metformin increased both AMPK activity and AMPK phosphorylation in VSMCs infected with adenovirus vector alone. However, metformin failed to activate AMPK if lncRNA-ANRIL was deficient in cells, demonstrating that metformin via lncRNA-ANRIL activates AMPK.

### Inhibition of lncRNA-ANRIL reduces the sensitivity of AMPK to AMP in cells

AICAR is an adenosine analog to generate AMP-mimetic action to activate AMPK [16]. We next infected cells with adenovirus containing lncRNA-ANRIL shRNA and then treated with metformin or AICAR, which did not affect the effects of lncRNA-ANRIL shRNA (Figure 3A). As illustrated in Figure 3B, 3C, AICAR or metformin alone increased AMPK phosphorylation at Thr172 and AMPK activity in VSMCs infected with adenovirus vector alone. This is consistent with previous reports [29, 30].

### Antisense oligonucleotide targeting lncRNA-ANRIL inhibits the effects of metformin on AMPK activation

To further implicate whether metformin via upregulating lncRNA-ANRIL activates AMPK, we used antisense oligonucleotide of lncRNA-ANRIL to inhibit the function of lncRNA-ANRIL. As shown in Figure 3D, antisense oligonucleotide of lncRNA-ANRIL reversed the effects on metformin in AMPK phosphorylation. We also observed that metformin increased AMPK phosphorylation in cells with overexpressed lncRNA-ANRIL (Figure 3E), demonstrating that metformin activates AMPK through upregulating lncRNA-ANRIL. The roles of lncRNA-ANRIL in PDGF-induced VSMC phenotypic switching were further examined by detecting the combination of PDGF and the lncRNA-ANRIL shRNA treatment on smooth muscle markers. As shown in Figure 3F–3I, PDGF not only decreased AMPK phosphorylation, but also altered the expression of α-SMA, calponin, and osteopontin in cells with overexpressed lncRNA-ANRIL shRNA.

### Metformin increases the binding of AMPKγ with lncRNA-ANRIL in VSMCs

**Figure 4:**
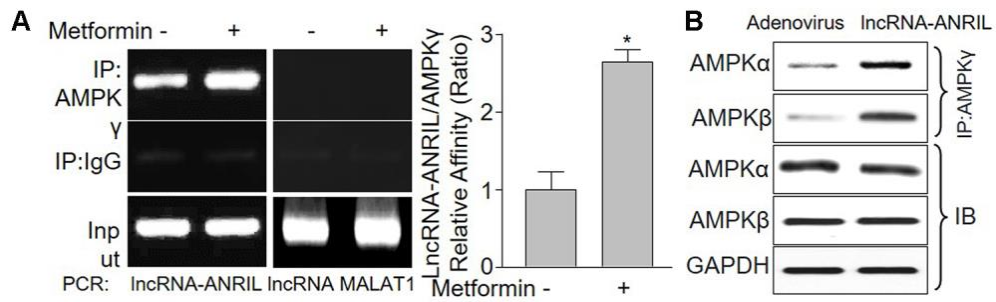
**LncRNA-ANRIL regulates AMPK catalytic activity by binding to AMPK gamma subunit.** (**A**) Cultured VSMCs were incubated with metformin (1 mM) for 12 hours. Cells were subjected to detect the binding of lncRNA-ANRIL or lncRNA-MALAT1 to AMPKγ subunit by using RNA-immunoprecipitation assay in (**A**) Quantitative analysis of the affinity between lncRNA-ANRIL and AMPKγ was performed. N is 5 in each group. ^*^P<0.05 vs. vehicle. (**B**) Cultured VSMCs were infected with adenovirus expressing lncRNA-ANRIL for 48 hours. The binding of AMPKα or β to AMPKγ was assayed by IP.

LncRNAs are associated to a plethora of cellular functions, most of which require the interaction with one or more RNA-binding proteins [31]. We next performed RIP analysis to determine the binding between AMPKγ and lncRNA-ANRIL. In Figure 4A, lncRNA-ANRIL, but not lncRNA-MALAT1, was positively detected in samples from cells following RIP with AMPKγ primary antibody, implying that lncRNA-ANRIL is specific to AMPKγ.

### LncRNA-ANRIL increases the formation of AMPKαβγ complex

To address what happens to AMPKγ subunit when it's bound to lncRNA-ANRIL, we detected the binding among AMPKα subunit, β subunit, and γ subunit. As shown in Figure 4B, the interaction among AMPKα, β and γ were much more solid in cells with lncRNA-ANRIL overexpression, compared with adenovirus-infected cells, indicating that lncRNA-ANRIL may increase the formation of AMPKαβγ complex.

### Metformin-induced AMPK activation is AMPKγ-dependent in cells

To further verify this concept that metformin activates AMPK through increasing the interaction between lncRNA-ANRIL and AMPKγ, we downregulated AMPKγ subunit via siRNA. As shown in Supplementary Figure 1A, 1B, metformin increased both AMPK phosphorylation and AMPK activity in VSMCs transfected with scramble siRNA, but not in cells transfected with AMPKγ siRNA. Collectively, it indicates that AMPKγ is essential for metformin-induced AMPK activation.

### Knockdown of lncRNA-ANRIL ablates the suppressive effects of AMPK activation by metformin on atherosclerotic plaque growth in *Apoe^-/-^* mice

**Figure 5:**
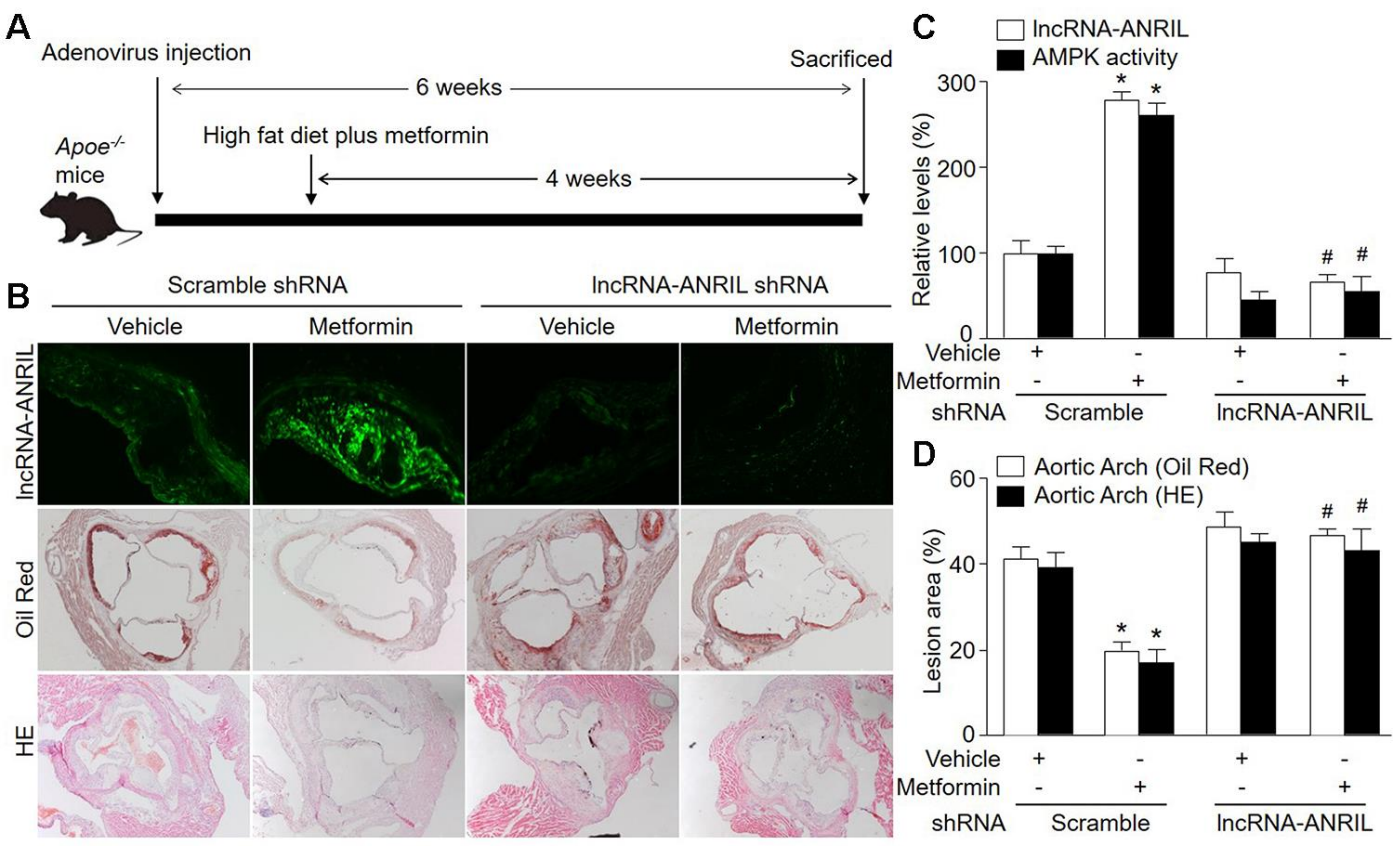
**Adenovirus-mediated knockdown of lncRNA-ANRIL prevents the anti-atherosclerotic effects of metformin in *Apoe^−/−^* mice.** (**A**) The protocol of animal experiments in *Apoe^−/−^* mice. Male *Apoe^−/−^* mice were injected with adenovirus containing scramble shRNA or lncRNA-ANRIL shRNA to silence lncRNA-ANRIL gene expression. Two weeks later after injection, mice received western diet plus metformin (150 mg/kg/day) in drinking water for 4 weeks. At the end of experiments, all mice were sacrificed under anesthesia. (**B**) The aortic root tissue was subjected to fluorescence in situ hybridization to detect lncRNA-ANRIL expression in green and the morphology analysis of aortic root by Oil Red staining and HE staining. (**C**) The expressional level of lncRNA-ANRIL and AMPK activity in aortic lesion tissue were assayed. (**D**) Quantitative analysis of atherosclerotic lesion size in aortic root by Oil Red staining and HE staining. N is 10-15 in each group. ^*^P<0.05 *vs.* scramble shRNA alone. ^#^*P*<0.05 vs. scramble shRNA plus metformin.

We further investigated if lncRNA-ANRIL is involved in the growth of atherosclerotic plaque suppressed by metformin *in vivo*. Thus, we infected *Apoe^-/-^* mice with adenovirus to knockdown lncRNA-ANRIL followed by feeding mice with western diet (Figure 5A). As indicated in Figure 5B, 5D, the size of atherosclerotic plaque was reduced in metformin-treated *Apoe^-/-^* mice expressing negative control shRNA, compared with vehicle-treated *Apoe^-/-^* mice expressing scramble shRNA. However, metformin did not decrease atherosclerotic plaque size in *Apoe^-/-^* mice infected with adenovirus containing lncRNA-ANRIL shRNA. According, metformin increased AMPK activity in atherosclerotic plaque in *Apoe^-/-^* mice expressing scramble shRNA, but not in *Apoe^-/-^* mice expressing lncRNA-ANRIL shRNA (Figure 5C). While, the plasms levels of TC, TG, glucose, and plasma cytokine levels were comparable among four groups (Supplementary Tables 2, 3). In sum, it indicates that lncRNA-ANRIL is involved in the effects of metformin in AMPK activation and atherosclerotic plaque growth *in vivo*.

### Knockdown of lncRNA-ANRIL abolishes the effects of metformin on VSMC phenotype switching *in vivo*

**Figure 6:**
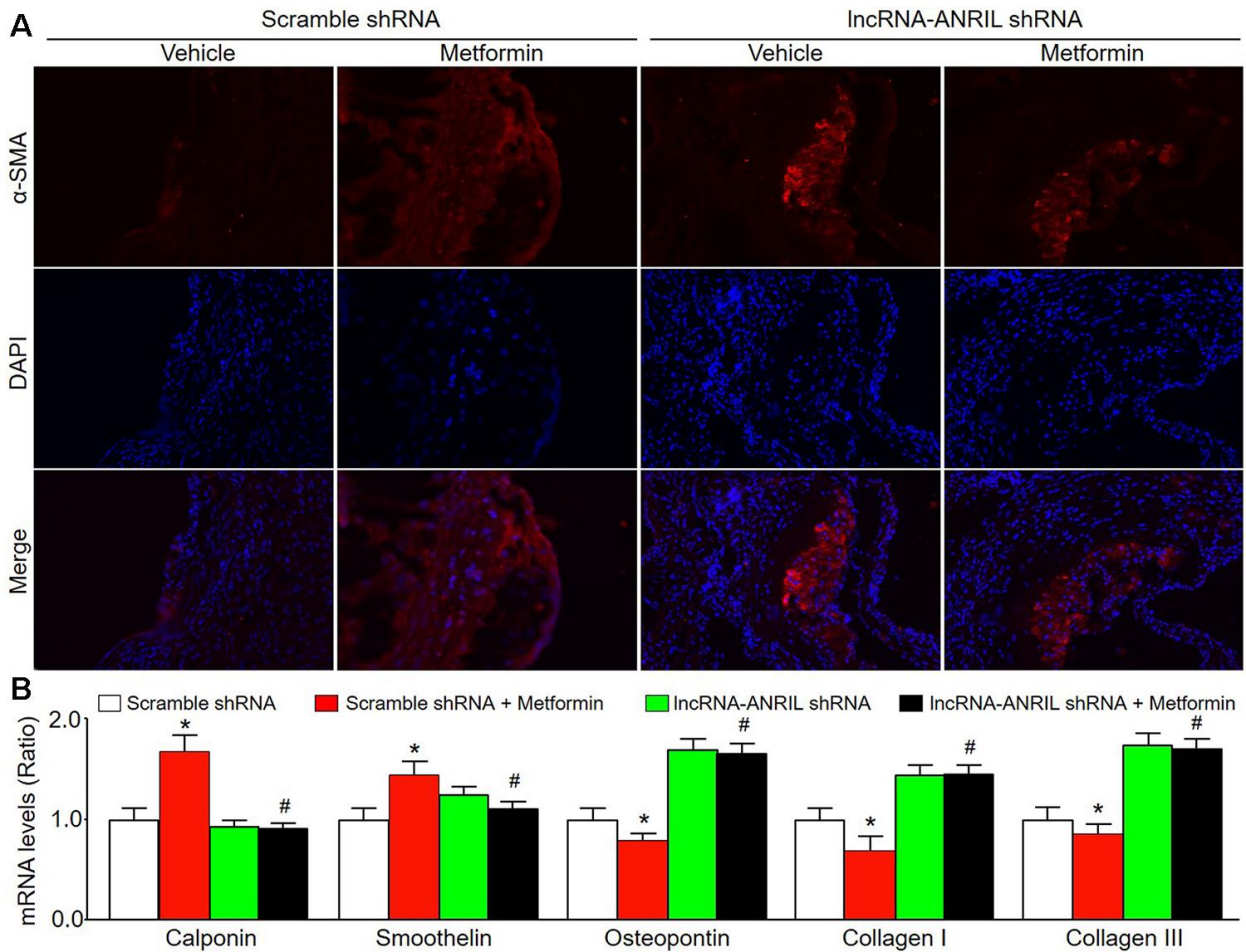
**Adenovirus-mediated knockdown of lncRNA-ANRIL prevents the effects of metformin on VSMC phenotype switch in *Apoe^−/−^* mice.** The animal experimental protocol was shown in Figure 5A. (**A**) The morphology of VSMC in atherosclerotic plaque was determined by immunofluorescence analysis of α-SMA. (**B**) The mRNA levels of the markers of VSMC phenotype switch, including calponin, smoothelin, osteopontin, collagen I, and collagen III in aortic tissue were measured by real-time PCR. N is 10-15 in each group. ^*^P<0.05 *vs.* scramble shRNA alone. ^#^*P*<0.05 vs. scramble shRNA plus metformin.

We next determined whether knockdown of lncRNA-ANRIL could induce VSMC phenotypic switching in atherosclerosis. As shown in Figure 6A, 6B, administration of metformin increased α-SMA content in atherosclerotic plaque in *Apoe^-/-^* mice fed with western diet infected with adenovirus expressing scramble shRNA. Meanwhile, metformin increased the mRNA levels of other contractile phenotypic markers (calponin and smoothelin) in these *Apoe^-/-^* mice. Conversely, the expressions of synthetic markers (osteopontin, collagen I, and collagen III) were markedly decreased by metformin in the plaque of *Apoe^-/-^* mice expressing scramble shRNA. However, metformin treatment had no any effects on the expression of markers including α-SMA, calponin, smoothelin, osteopontin, collagen I, and collagen III in the plaque of *Apoe^-/-^* mice expressing lncRNA-ANRIL shRNA. Taken together, these results suggest that metformin via lncRNA-ANRIL upregulation suppresses VSMC phenotypic switching in atherosclerosis.

### Reduced AMPK phosphorylation, AMPK activity, and lncRNA-ANRIL expression are associated with atherosclerotic lesions in human patients

To establish the clinical association of this study, we determined the level of AMPK activity and AMPK phosphorylation in patients with atherosclerosis. The clinical characters were presented in Supplementary Table 4. As shown in Supplementary Figure 2A, the plaques were seen in atherosclerotic samples (stenosis > 50%). The atherosclerotic tissues exhibited lower levels of AMPK phosphorylation and activity than control samples (Supplementary Figures 2B, 2C). Further, the expression of lncRNA-ANRIL was also decreased in patient with atherosclerosis (Supplementary Figure 2D).

## DISCUSSION

In this study, we not only identified lncRNA-ANRIL as an AMPK regulator, but also demonstrated that metformin via lncRNA-ANRIL-dependent AMPK pathway prevents VSMC phenotype switches in the development of atherosclerotic plaque. In cultured cells, metformin upregulates lncRNA-ANRIL expression to increase the interaction between lncRNA-ANRIL and AMPKγ subunit. In mice, AMPK activation by metformin suppressed the growth of atherosclerotic lesion. Importantly, all effects of AMPK activation by metformin were abolished by knockdown of lncRNA-ANRIL via genetic approaches. Thus, we thought that lncRNA-ANRIL upregulation is essential for metformin-suppressed atherosclerosis.

The major discovery of this study is that we identified lncRNA-ANRIL as a novel modulator of AMPK. This is supported by following evidence: (1) The specific binding of AMPKγ to lncRNA-ANRIL was detectable using RIP analysis; (2) LncRNA-ANRIL knockdown decreased metformin-induced AMPK phosphorylation and AMPK activation; (3) The effects of metformin, as a well-recognized AMPK activator, were abolished by lncRNA-ANRIL deficiency *in vitro* and *in vivo.* Although at least two upstream kinases including Calmodulin-dependent kinase kinase (CaMKK) and liver kinase B1 (LKB1) have been identified as AMPK kinases [32, 33], we firstly showed that lncRNA-ANRIL regulates AMPK activity by direct interaction. Linking many functions of AMPK, for example, glucose and lipid metabolisms [34], the finding that lncRNA-ANRIL is an AMPK regulator may expand the applications of lncRNA-ANRIL.

Another important finding is that metformin via lncRNA-ANRIL inhibits VSMC phenotypic switching and the growth of atherosclerotic plaque. Hyosuk Cho et al have identified and validated ten target genes downstream of ANRIL [13]. In this study, we not only identified AMPK as a new target of lncRNA-ANRIL, but also demonstrated that lncRNA-ANRIL/AMPK pathway is critical in the progression of atherosclerosis. Our observations demonstrate that dysregulation of lncRNA-ANRIL in VSMCs affects critical cellular functions by alternating the expressional levels of some key genes of VSMC phenotypic switching.

An issue remained in this study is the signaling pathway by how lncRNA-ANRIL increases APMK activity. Based on our observations, we thought this binding between lncRNA-ANRIL and AMPKγ may increase the stability of AMPKαβγ complex. This wound increase the sensitivity of AMP to AMPK complex and preserve the phosphorylation of AMPKα at Thr172 because we found the formation of AMPKαβγ was increased by lncRNA-ANRIL overexpression and the sensitivity of AICAR to AMPK was also enhanced.

In summary, we identify a novel mechanism by which metformin via lncRNA-ANRIL pathway limits VSMC phenotypic switching, resulting in the suppression of atherosclerosis lesion (Supplementary Figure 3). Because loss of lncRNA-ANRIL can effectively attenuate the effects of AMPK activation, both AMPK and lncRNA-ANRIL might be a therapeutic target on the prevention of atherosclerosis-associated vascular diseases, such as stroke.

## MATERIALS AND METHODS

Expanded Materials and Methods are available in Supplementary Material.

### Cell culture

As described previously [29], primary murine VSMCs were grown in Smooth Muscle Cell Medium (Sciencell, USA). Cells between passages 4 and 8 were used.

### Western blot analysis

Total 20 μg protein were subjected to western blot analysis as described previously [35].

### RNA-immunoprecipitation (IP) assay

As described previously [36], lysates were incubated in cold room overnight with magnetic protein A/G beads pretreated with antibody. RNA was extracted from IP complex. The PCR primers for lncRNA-ANRIL are shown in Supplementary Table 1.

### Animal experimental protocol

Male *Apoe^-/-^* mice were obtained from Beijing Huafukang Animal Experimental Center. Mice received tail vein injection of adenovirus. Two weeks after injection, mice were fed with western diet plus metformin (150 mg/kg/day) in drinking water for 4 weeks. At the end of experiments, mice were sacrificed under anesthesia by intraperitoneal injection of 0.8% pentobarbital sodium (60 mg/kg).

### Atherosclerotic lesion analysis

As described previously [37], Oil Red staining was used for neutral lipids in atherosclerotic lesion.

### AMPK activity assay

The SAMS peptide was used to assay AMPK activity as previously described [38, 39].

### Statistical analysis

All data are expressed as mean ± S.E.M. Multiple comparisons were performed using a one-way ANOVA followed by Bonferroni corrections. Two-sided *P* < 0.05 was considered significant.

## Supplementary Material

Supplementary Materials and Methods

Supplementary Figures

Supplementary Tables
