# Supplementary material for: Overexpression of long noncoding RNA ANRIL inhibits phenotypic switching of vascular smooth muscle cells to prevent atherosclerotic plaque development in vivo
Source: Aging (Albany NY). 2020 Dec 19;13(3):4299–316. doi: 10.18632/aging.202392 (PMC7906209; doi:10.18632/aging.202392)
Supplement: Supplementary Materials and Methods [file aging-13-202392-s001.pdf]

SUPPLEMENTARY MATERIALS AND METHODS

Reagents and animals

Polyclonal or monoclonal antibodies against pAMPKα-Thr172, AMPKα, AMPKγ1, and α-SMA were obtained from Santa Cruz Biotechnology or Cell Signaling Company. All secondary antibodies were from Cell Signaling Technology. AMPKγ1 siRNA was from Santa Cruz. All PCR primers were purchased from Invitrogen. Other chemicals, if not indicated, were from Sigma-Aldrich (St. Louis, MO). Male *Apoe*$^{-/-}$ mice were obtained from Beijing Huafukang Animal Experimental Center. Mice were housed in temperature-controlled cages with a 12-hrs light-dark cycle. The animal protocol was reviewed and approved by the University of Central South Animal Care and Use Committee.

Cell culture

Primary murine vascular smooth muscle cells (VSMCs) isolated from mice were grown in Smooth Muscle Cell Medium (Sciencell, USA) supplemented with 2% fetal bovine serum, penicillin (100U/ml) and streptomycin (10mg/ml) as described previously [1]. In all experiments, cells were used between passages 4 and 8. All cells were incubated at 37° C in a humidified atmosphere of 5% CO_2 and 95% air. Cells were grown to 80% confluency before being treated with different agents.

Transfection of siRNA into cells

Transient transfection of siRNA was carried out according to Santa Cruz's protocol [2]. Briefly, the siRNAs were dissolved in siRNA buffer (20 mM KCl; 6 mM HEPES, pH 7.5; 0.2 mM $MgCl_2$) to prepare a 10 µM stock solution. Cells grown in 6 well plates were transfected with siRNA in transfection medium (Gibcol) containing liposomal transfection reagent (Lipofectamine RNAimax, Invitrogen). For each transfection, 100 µl transfection medium containing 4 µl siRNA stock solution was gently mixed with 100 µl transfection medium containing 4 µl transfection reagent. After 30-min incubation at room temperature, siRNA-lipid complexes were added to the cells in 1.0 ml transfection medium, and cells were incubated with this mixture for 6 h at 37° C. The transfection medium was then replaced with normal medium, and cells were cultured for 48 hours.

Generations of DNA constructs

LncRNA-ANRIL cDNA or shRNA was purchased from Origene Company. The adenovirus construction compassing lncRNA-ANRIL cDNA or shRNA was generated using the AdMax (Microbix) and pSilencer™ adeno 1.0-CMV (Ambion) systems according to the manufacturers' recommendations. Viruses were packaged and amplified in HEK293A cells and purified using $CsCl_2$ banding followed by dialysis against 10 mM Tris-buffered saline with 10% glycerol. Titering was performed on HEK293 cells using the Adeno-X Rapid Titer kit (BD Biosciences Clontech, Palo Alto, CA, USA) according to the manufacturer's instructions.

Adenovirus infections to cells or animals

Cells were infected with adenovirus overnight in antibiotics-free medium supplemented with 2% FBS. The cells were then washed and incubated in fresh medium for an additional 12-hour before experiments. For infecting mice, adenovirus was injected via tail vein under pressure in 100µl of PBS with 7.6 X 10^7 IFUs of loaded virus. The concentration of DNA was 10 mg/kg.

Western blot analysis

Cell lysates or tissue homogenates were subjected to western blot analysis, as described previously [3]. The protein content was assayed by BCA protein assay reagent (Pierce, USA). Protein of 20 µg was loaded to SDS-PAGE and then transferred to membrane. Membrane was incubated with a 1:1000 dilution of primary antibody, followed by a 1:5000 dilution of horseradish peroxidase-conjugated secondary antibody. Protein bands were visualized by ECL (GE Healthcare). The intensity (area X density) of the individual bands on Western blots was measured by densitometry (model GS-700, Imaging Densitometer; Bio-Rad).

RNA-immunoprecipitation (IP) assay

The Magna RIP kit was used for RNA IP assay as described previously [4]. Briefly, whole-cell lysates were incubated at 4° C overnight with magnetic protein A/G beads pretreated with 5 µg rabbit IgG or AMPK gamma antibody. Beads were washed and incubated with proteinase K buffer (30 min at 55° C), then RNA was isolated from immunoprecipitates, and cDNA was synthesized. lncRNA-ANRIL cDNA was amplified by PCR.

RNA quantifications by RT-qPCR

Total RNA was isolated using a TRIzol-based (Invitrogen) RNA isolation protocol. For mRNA detections, the iScript cDNA Synthesis Kit (Bio-Rad) was used to synthesize first-strand cDNA according to

the manufacturer's protocol [5]. Reactions were run for 30 cycles at conditions as follows: denaturation for 30 seconds at 94° C, annealing for 30 seconds at 57° C, and extension for 30 seconds at 72° C. Constitutively expressed GADPH mRNA was amplified as control. All primers were presented in supplementary Table 1.

Animal experimental protocols

Mice received tail vein injection of adenovirus containing scramble shRNA or lncRNA-ANRIL shRNA. Two weeks later after injection, mice were received western diet with or without metformin (150 mg/kg/day) in drinking water for 4 weeks. At the end of experiments, all mice were sacrificed under anesthesia by intraperitoneal injection of 0.8% pentobarbital sodium (60 mg/kg).

Atherosclerotic lesion analysis

As described previously [6], after being fed the Western diet for 4 weeks, the mice were fasted for 14 h and then were anesthetized and euthanized. The heart and aortic tissue were removed from the ascending aorta to the ileal bifurcation and placed in 4% paraformaldehyde for 16 h. After fixation, the adventitia was thoroughly cleaned under a dissecting microscope. For analyzing the lesion area in the aortic root, the heart was dissected from the aorta, embedded in Polyfreeze tissue freezing medium (Polysciences, Inc) and sectioned (5 µm thickness). Four consecutive sections were collected from each mouse and stained with Oil Red O for neutral lipids, and counterstained with hematoxylin to visualize the nuclei. Plaques were captured under the Olympus microscope connected to a QImaging Retiga CCD camera. The aortic lesion size of each animal was obtained by the averaging of lesion areas in four sections from the same mouse. Digital images of the aorta were captured under a stereomicroscopy, and the lesion area was quantified from the aortic arch to 5 mm distal of the left subclavian artery using Alpha Ease FC software (version 4.0 Alpha Innotech).

Immunofluorescence (IFC)

As described previously [7], after treatment, cells on sterile glass cover slips were rinsed by cold PBS and then fixed by incubation with 10% formalin in PBS for 10 minutes. Block cells by 5% BSA for 30 minutes. Incubate cells with primary antibody for 1 hour at room temperature or overnight at 4° C. After washing, incubate with fluorescence-conjugated secondary antibody for 45 minutes. Take picture in fluorescence microscope.

Detection of lncRNA-ANRIL by fluorescence in situ hybridization (FISH)

As described previously with minor modifications [5], FISH of lncRNA-ANRIL was performed on 5-µm paraffin embedded arterial sections. Briefly, paraffin embedded tissue sections were cut using a microtome (Leica, RM2235) and mounted on polylysine microscope slides (Fisher Scientific) and stored at room temperature (RT) until FISH. Paraffin wax was removed in xylene, sections rehydrated in a series of decreasing ethanol solutions and washed with PBS before fixing in 4% paraformaldehyde. To block endogenous peroxide activity, tissue sections were treated with 0.3% H_2O_2 and washed in PBS before acetylating in acetic anhydride/triethanolamine. Sections were then washed in 2 X SSC and PBS before permeabilization with proteinase K (5 µg/ml) and washes with PBS. Probes (5' and 3'-DIG labelled LNA miRCURY probes; Exiqon) were denatured at 90° C before dilution in hybridization buffer (50% formamide, 0.3 M NaCl, 20 mM Tris-HCL, 5 mM EDTA, 10 mM $NaPO_4$, 10% Dextran sulphate, 1 X Denhardt's solution, 0.5 mg/ml yeast tRNA). Tissue sections were hybridized with lncRNA-ANRIL (200 nM) overnight at 21° C below the predicted Tm value of the probe. After post-hybridization washes in 5 X SSC at RT; 50% formamide/1 X SSC/0.1% Tween 20 at the hybridization temperature; 0.2 X SSC at RT, FISH signals were detected using an Anti-Digoxigenin antibody and the tyramide signal amplification system (PerkinElmer) according to the manufacturer's instructions. Tissue sections were mounted in Vectashield (Vector laboratories). All fluorescence images were analyzed with a Nikon TE2000-U inverted microscope.

AMPK activity assay

AMPK activity was assayed by using the SAMS peptide as previously described [8]. Briefly, duplicate tubes with 200 µg of protein from each sample were prepared and mixed with 500 µl of IP buffer (lysis buffer plus 1 mM dithiothreitol). AMPK was then immunoprecipitated by adding 10 µg of polyclonal antibody against AMPK and 25 µl of Protein A/G-agarose and incubated at 4° C. After centrifugation (14,000 *g*, 1 min), the beads were washed with IP buffer and then twice with 10X reaction buffer (400 mM HEPES, pH 7.4, 800 mM NaCl, 50 mM $MgCl_2$, 1 mM dithiothreitol). The AMPK activity was assayed by adding 50 µl of reaction mixtures, consisting of 5 µl of reaction buffer, 10 µl of SAMS peptide (1 mg/ml), 10 µl of ATP working stock consisting of 0.1 µl of 100 mM ATP, 1 µl of ^{32}P-ATP, and 8.9 µl of H_2O, 25 µl H_2O, or 25 µl of 400 µM AMP, and incubated at 37° C for 10 min. The beads were quickly pelleted, and 25 µl of supernatant was spotted onto P81 Whatman paper. The

filter papers were then washed four or five times with 1% phosphoric acid. After the final wash, the filters were quickly dried and counted in a scintillation counter. The difference between the presence and absence of AMPK is calculated as the AMPK activity.

Measurements of blood glucose, cholesterol, triglyceride, and plasma cytokine levels

The determinations of blood glucose, cholesterol, triglyceride, and plasma cytokine levels in serum were assayed using commercial kits, which were accordance with the protocols as described previously [7].

Patients and sample processing

Left anterior descending coronary arteries were obtained from human subjects after sudden death. The demographic data were presented in Supplementary Table 3. The histology of left anterior descending coronary artery was determined by HE staining. Atherosclerosis was diagnosed as the amount of stenosis of lumen was over 100%. The study was conducted in accordance with the principles of Good Clinical Practice and the Declaration of Helsinki. The study protocol was approved by the Ethical Committee of Central South University Xiangya Hospital, and informed consent was obtained from each human subject.

Statistical analysis

All quantitative results are expressed as mean ± SEM. The normal distribution of data was tested by the Kolmogorov-Smirnov test before statistical comparisons, and the normality/equal variance was tested to determine whether ANOVA was appropriate. All data were analyzed with a 1-way ANOVA followed by Bonferroni *post-hoc* analyses. Comparisons between two groups were analyzed by unpaired Student's t test between two groups. Statistical analysis was conducted using IBM SPSS statistics 20.0 (IBM Corp., Armonk, NY, USA), and a two-sided P-value < 0.05 was considered significant.

REFERENCES

1. Wang S, Zhang C, Zhang M, Liang B, Zhu H, Lee J, Viollet B, Xia L, Zhang Y, Zou MH. Activation of AMP-activated protein kinase α2 by nicotine instigates formation of abdominal aortic aneurysms in mice in vivo. Nat Med. 2012; 18:902–10. https://doi.org/10.1038/nm.2711 PMID:22561688

2. Wang S, Xu J, Song P, Wu Y, Zhang J, Chul Choi H, Zou MH. Acute inhibition of guanosine triphosphate cyclohydrolase 1 uncouples endothelial nitric oxide synthase and elevates blood pressure. Hypertension. 2008; 52:484–90. https://doi.org/10.1161/HYPERTENSIONAHA.108.112094 PMID:18645049

3. Wang S, Xu J, Song P, Viollet B, Zou MH. In vivo activation of AMP-activated protein kinase attenuates diabetes-enhanced degradation of GTP cyclohydrolase I. Diabetes. 2009; 58:1893–901. https://doi.org/10.2337/db09-0267 PMID:19528375

4. Li J, Gong L, Liu S, Zhang Y, Zhang C, Tian M, Lu H, Bu P, Yang J, Ouyang C, Jiang X, Wu J, Zhang Y, et al. Adipose HuR protects against diet-induced obesity and insulin resistance. Nat Commun. 2019; 10:2375. https://doi.org/10.1038/s41467-019-10348-0 PMID:31147543

5. Bai YP, Zhang JX, Sun Q, Zhou JP, Luo JM, He LF, Lin XC, Zhu LP, Wu WZ, Wang ZY, Zhang GG. Induction of microRNA-199 by nitric oxide in endothelial cells is required for nitrovasodilator resistance via targeting of prostaglandin I2 synthase. Circulation. 2018; 138:397–411. https://doi.org/10.1161/CIRCULATIONAHA.117.029206 PMID:29431644

6. Ding Y, Zhang M, Zhang W, Lu Q, Cai Z, Song P, Okon IS, Xiao L, Zou MH. AMP-activated protein kinase alpha 2 deletion induces VSMC phenotypic switching and reduces features of atherosclerotic plaque stability. Circ Res. 2016; 119:718–30. https://doi.org/10.1161/CIRCRESAHA.116.308689 PMID:27439892

7. Li P, Yin YL, Guo T, Sun XY, Ma H, Zhu ML, Zhao FR, Xu P, Chen Y, Wan GR, Jiang F, Peng QS, Liu C, et al. Inhibition of aberrant MicroRNA-133a expression in endothelial cells by statin prevents endothelial dysfunction by targeting GTP cyclohydrolase 1 in vivo. Circulation. 2016; 134:1752–65. https://doi.org/10.1161/CIRCULATIONAHA.116.017949 PMID:27765794

8. Wang S, Liang B, Viollet B, Zou MH. Inhibition of the AMP-activated protein kinase-α2 accentuates agonist-induced vascular smooth muscle contraction and high blood pressure in mice. Hypertension. 2011; 57:1010–17. https://doi.org/10.1161/HYPERTENSIONAHA.110.168906 PMID:21464390
