# Supplementary material for: Overexpression of long noncoding RNA ANRIL inhibits phenotypic switching of vascular smooth muscle cells to prevent atherosclerotic plaque development in vivo
Source: Aging (Albany NY). 2020 Dec 19;13(3):4299–316. doi: 10.18632/aging.202392 (PMC7906209; doi:10.18632/aging.202392)
Supplement: Supplementary Figures [file aging-13-202392-s002.docx]

SUPPLEMENTARY FIGURES


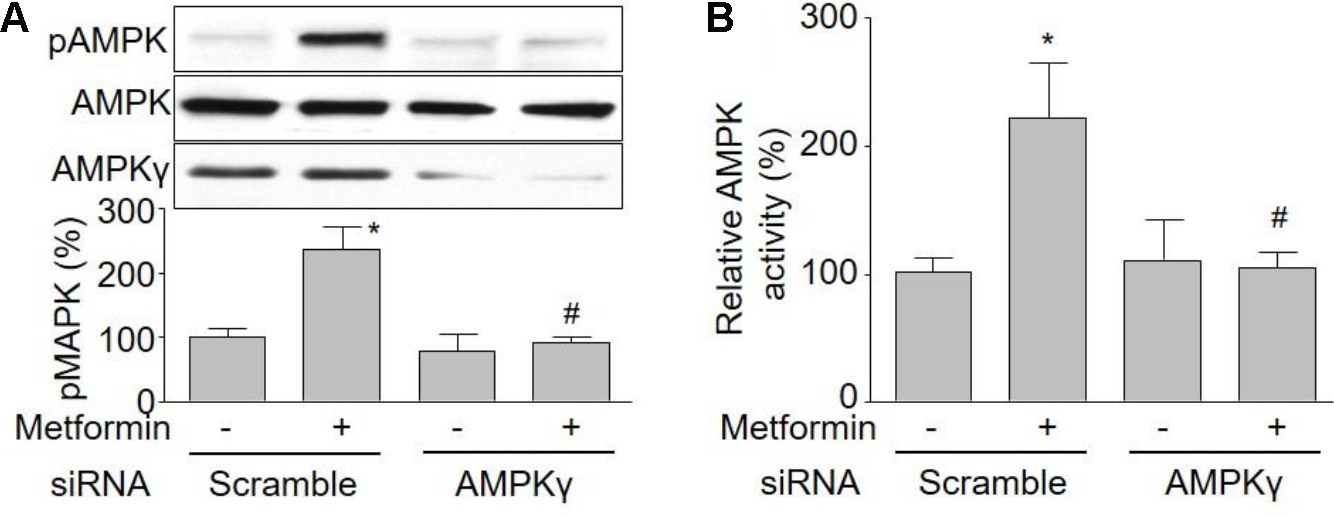


Supplementary Figure 1. LncRNA-ANRIL regulates AMPK catalytic activity by AMPK gamma subunit. Cultured VSMCs were transfected with AMPKγ1 siRNA for 48 hours followed by treatment with metformin (1 mM) for 12 hours. Total cell lysates were subjected to perform western blot analysis of phosphorylated AMPK (pAMPK) and total AMPK protein levels in (A). AMPK activity was assayed by P^32^-ATP method in (B). N is 5 in each group. ^*^*P*<0.05 vs. scramble siRNA. ^#^*P*<0.05 vs. scramble siRNA plus metformin.


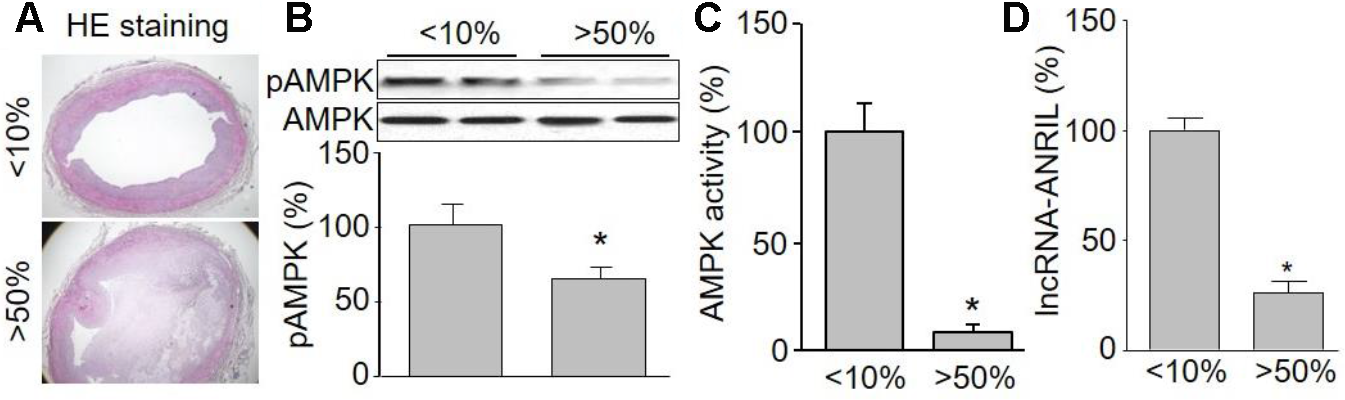


Supplementary Figure 2. Decreased AMPKα phosphorylation and AMPK activity is related to the development of atherosclerosis lesions in human subjects. Tissues of left anterior descending coronary artery were collected from patients with atherosclerosis diagnosed by coronary angiography. (A) Morphology of atherosclerosis lesion by HE staining (X100) and IHC analysis of pAMPK and AMPK (X400). (B) Quantitative data for pAMPK in A. (C) The homogenates of atherosclerotic tissues were subjected to assay the level of pAP-2α by Western blot. (D) The lncRNA-ANRIL level was assessed by real-time PCR. Five human subjects in each group. *P<0.05 vs. 10%.


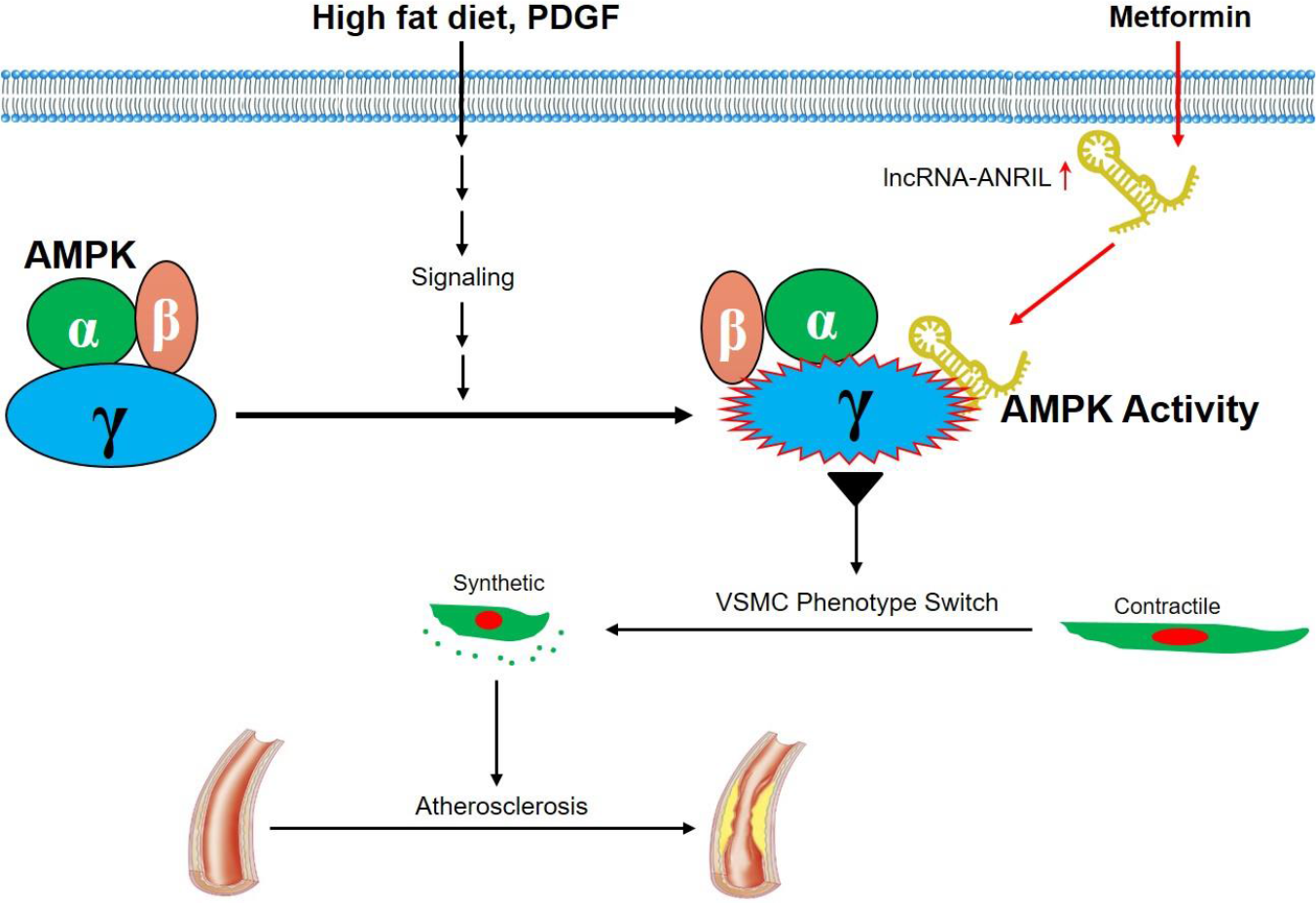


Supplementary Figure 3. Proposed mechanisms by which metformin prevents atherosclerosis. AMPK activation by metformin increases lncRNA-ANRIL expression, leading to the increased affinity of lncRNA-ANRIL to AMPKγ subunit. As a result, AMPK catalytic activity is increased. In this way, metformin produces the suppressive effects on the development of atherosclerosis through suppression of VSMC phenotypic switching.
