# Supplementary material for: Overexpression of long noncoding RNA ANRIL inhibits phenotypic switching of vascular smooth muscle cells to prevent atherosclerotic plaque development in vivo
Source: Aging (Albany NY). 2020 Dec 19;13(3):4299–316. doi: 10.18632/aging.202392 (PMC7906209; doi:10.18632/aging.202392)
Supplement: Supplementary Tables [file aging-13-202392-s003.pdf]

SUPPLEMENTARY TABLES

Supplementary Table 1. Primers used in this project.

Gene	Forward primer sequence (5' - 3')	Reverse primer sequence (5' - 3')
Calponin	GGCAACCAACCACAAATTAGCA	CCCTGCATGCGGTGGAAAAGGC
Smoothelin	GGGGCTCTTGGGAACTACAC	GCCATGAATGTCCACTGTGC
α-SMA	AATGCCACCTTTTGACAGTGATG	TGGAATGTCAAGTCTGCACCA
Collagen I	TGCTTGAGGTGGTTGTGGAA	CAGCAACTGTGACCTGGAGA
Collagen I	CTGATGATCGACCGTGAGAA	GAAGGGTTGGTTCTTTCGAA
Osteopontin	AACTTGGGTTTCAGCACCAC	TACCAGTCCCACGATGTCAG
lncRNA-ANRIL	GTCACTGCAGCTCTGAATGTTTCTT	CCCAGTTGTGCATCGACCTA
GAPDH	AGCTAAGAGAAGGGCGGAAC	CATCTGCAGGCTGACATTGA

Supplementary Table 2. Serum lipid and glucose levels in *Apoe*$^{-/-}$ mice.

Groups	Cholesterol (mM)	Triglyceride (mM)	Glucose (mg/dl)
Scramble shRNA	15.47±3.54	1.07±0.19	168±23
Scramble shRNA+Metformin	14.10±3.24	0.98±0.18	170±30
lncRNA-ANRIL shRNA	13.53±2.98	1.17±0.21	167±31
lncRNA-ANRIL shRNA+Metformin	13.27±3.43	0.95±0.13	161±23

Male *Apoe*$^{-/-}$ mice were infected with adenovirus via injection of tail vein. Two weeks later, mice were fed with western diet and received metformin in drinking water for 4 weeks. At the end of experiments, all mice were sacrificed under anesthesia and serum levels of cholesterol, triglyceride, and glucose in all fasting mice were assayed. N is 10-15 in each group.

Supplementary Table 3. Plasma cytokine levels in *Apoe*$^{-/-}$ mice.

Groups	IL-1β (pg/ml)	IL-6 (pg/ml)
Scramble shRNA	166.21±8.19	134.62±5.86
Scramble shRNA+Metformin	157.64±6.55	137.13±8.01
lncRNA-ANRIL shRNA	148.73±7.16	126.32±6.68
lncRNA-ANRIL shRNA+Metformin	140.69±8.23	130.63±7.38

Male *Apoe*$^{-/-}$ mice were infected with adenovirus via injection of tail vein. Two weeks later, mice were fed with western diet and received metformin in drinking water for 4 weeks. At the end of experiments, all mice were sacrificed under anesthesia and serum levels of IL-1β and IL-6 were assayed. N is 10-15 in each group.

Supplementary Table 4. Demographic data for individuals with or without atherosclerosis.

ID	Gender	Ages (years old)	Stenosis (%)
1	Male	47	8%
2	Male	46	7%
3	Female	59	10%
4	Female	26	5%
5	Male	47	7%
6	Female	57	22%
7	Male	40	39%
8	Female	46	14%
9	Male	51	27%
10	Male	62	41%
11	Male	74	98%
12	Male	59	82%
13	Female	43	63%
14	Female	68	77%
15	Male	54	86%
